# Stack-VTP: prediction of vesicle transport proteins based on stacked ensemble classifier and evolutionary information

**DOI:** 10.1186/s12859-023-05257-5

**Published:** 2023-04-07

**Authors:** Yu Chen, Lixin Gao, Tianjiao Zhang

**Affiliations:** College of Information and Computer Engineering, Northeast Forestry University, Harbin, China

**Keywords:** Protein prediction, Vesicle transport proteins, Ensemble learning, Stacked model

## Abstract

Vesicle transport proteins not only play an important role in the transmembrane transport of molecules, but also have a place in the field of biomedicine, so the identification of vesicle transport proteins is particularly important. We propose a method based on ensemble learning and evolutionary information to identify vesicle transport proteins. Firstly, we preprocess the imbalanced dataset by random undersampling. Secondly, we extract position-specific scoring matrix (PSSM) from protein sequences, and then further extract AADP-PSSM and RPSSM features from PSSM, and use the Max-Relevance-Max-Distance (MRMD) algorithm to select the optimal feature subset. Finally, the optimal feature subset is fed into the stacked classifier for vesicle transport proteins identification. The experimental results show that the of accuracy (ACC), sensitivity (SN) and specificity (SP) of our method on the independent testing set are 82.53%, 0.774 and 0.836, respectively. The SN, SP and ACC of our proposed method are 0.013, 0.007 and 0.76% higher than the current state-of-the-art methods.

## Introduction

Protein is an important raw material for building and repairing the human body, and it can also provide energy for the human body’s life activities. It has a variety of functions in different cell cavities of eukaryotic cells [1]. Vesicle transport proteins are one of the most important proteins and play an important role in facilitating the vesicle transport process. Vesicle transport proteins assist vesicular transport activities that occur widely within and between cells, such as neurotransmitter transport between nerve cells, protein transport between the endoplasmic reticulum and the Golgi apparatus, and transport from the Golgi apparatus to lysozymes body, secretory vesicles, etc. Vesicle transport proteins are also of great importance in the biomedical field. Aberrant vesicle transport proteins have contributed to multiple human diseases [2], such as chylomron retention disease [3] and Hermansky-Pudlaksyndrome.

Since vesicle transport proteins play an important role in the function and structure of eukaryotic cells, and their relationship with diseases is becoming more and more clear, the in-depth study of vesicle transport proteins is particularly significant. In the field of biological research, researchers have achieved certain result in the identification of vesicle transport proteins, such as morpholino knockdown [4] and dissection [5]. However, traditional identification methods are very expensive and time-consuming. In recent years, computer-aided methods have been developed to rapidly and accurately identify vesicular transport proteins. Andersson and Sandelius [6] applied a web-based subcellular prediction tool to search the Arabidopsis genome for homologues of chloroplast-localized cytoplasmic vesicle trafficking components. Lindquist et al. [7] conducted bioinformatic analysis to pinpoint the role of two common vesicle transport proteins (Coat and Clathrin). Le et al. [8] adopted Gate Recurrent Unit (GRU) to build a model to classify the molecular functions of Rab GTPases in the vesicular transport system. Tao et al. [9] used MRMD and LibSVM to identify vesicle transport proteins. Gong et al. [10] proposed the VTP-Identifier model, which selected PSSM as feature and adopted XGBoost as classifier to identify vesicle transport proteins.

Although some achievements have been made, there are still some limitations of current method. On the one hand, traditional single machine learning methods have their own biases, which cannot comprehensively learn protein-related features, and only perform well in specific proteins. On the other hand, deep learning methods achieve a certain level of accuracy but are relatively time-consuming and computation-expensive. In order to make up for the above shortcomings, we propose to apply an integrated strategy to construct a classifier to identify vesicle transport proteins.

The idea of ensemble learning is to combine several sub-learners through a certain strategy to generate a strong learner. In recent years, ensemble learning has become one of the research hotspots in the field of computer science and application, which has attracted the attention of many scholars. Kearns [11] studied the equivalence between weak learning algorithm and strong learning algorithm in learning model. Schapire [12] explored the feasibility of combining multiple weak models into a high-precision model. Nguyen et al. [13] proposed a variational inference method for multivariate Gaussian distribution estimation and a combination algorithm adaptive method based on evolutionary computation. In addition to being widely concerned in the computer science and application field, ensemble learning has also been widely used in bioinformatics, computer-aided diagnosis and computer vision. Zhang et al. [14] integrated the LightGBM model of learning a single feature into a unified ensemble framework and constructed a two-layer integration model to identify non-classical secreted proteins. Chen et al. [15] proposed a method for identifying moonlighting proteins based on bagging-SVM. They firstly extracted SVMProt-188D features from protein sequences, then applied linear discriminant analysis for feature selection, and finally used bagging-SVM to accurately identify noonlighting proteins. Zheng et al. [16] developed a fully convolutional network based meta-learner to learn how to improve the basic learner, and constructed a new ensemble learning framework for 3D biomedical image segmentation.

Inspired by previous research, in this study, we construct a stacked ensemble model called Stack-VTP to identify vesicle transport proteins. Firstly, we preprocess the imbalanced data by random undersampling. Secondly, PSSM is extracted from the protein sequences, followed by further AADP-PSSM and RPSSM features from the PSSM, and the optimal feature subset is selected using MRMD algorithm while removing irrelevant features to reduce the feature dimensionality. Finally, a two-layer stacked classifier is constructed to identify vesicle transport proteins. We are the first to propose a stacked ensemble strategy to construct classifiers to identify vesicle transport proteins, solving the problem that traditional machine learning methods are biased and deep learning takes a long time. Our method not only achieve better results in the identification of vesicle transport proteins than before, but also provide a new idea for researchers to combine integrated strategies with evolutionary information features to identify proteins. Furthermore, our study aids in the design of therapeutic agents for diseases related to vesicle transport proteins, and the determination of vesicle transport protein abnormalities.

## Materials and methods

**Fig. 1:**
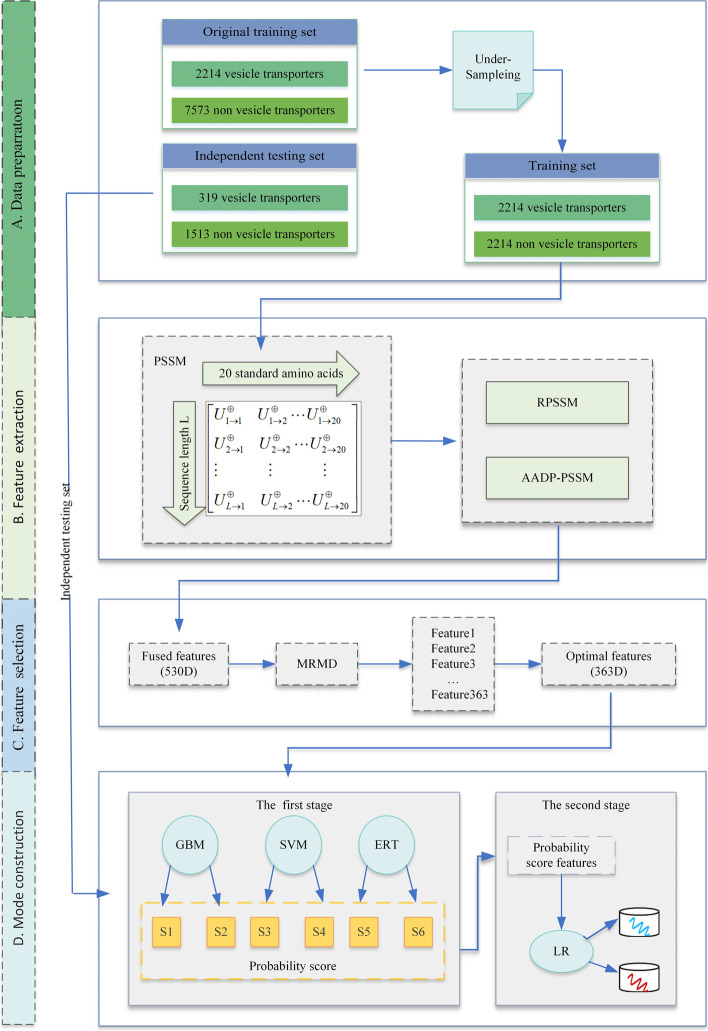
The flowchart of the vesicle transport proteins prediction model

The study is divided into four parts and the pipeline is shown in Fig. 1. Firstly, the dataset of Le et al. is used as the benchmark dataset, and the dataset is divided into two parts, the training set and the independent testing set, and the training set is undersampled to solve the data imbalance problem(A). Next, we extract PSSM features from the protein sequences and further extract RPSSM and AADP-PSSM features from the PSSM (B). Then, we use MRMD algorithm to reduce the dimensionality and obtain the optimal feature subset (C). Finally, base classifiers and meta-classifiers are selected from multiple traditional machine learning classifiers to construct a stacked model (D).

### Benchmark dataset

In this study, we use the dataset of Le et al. [8] as the benchmark dataset. The numbers of vesicular and non-vesicular transport proteins are 2533 and 9086, respectively, and we define vesicular transportation proteins as positive samples and non-vesicular transport proteins as negative samples. We divide the dataset into a training set and an independent testing set, as shown in Table 1.

**Table 1:** Statistical information on the dataset in this study

	Original	Identity<30%	Training set	Testing set
Vesiclele transport proteins	7108	2533	2214	319
Non-vesicle transport proteins	17,656	9086	7573	1513

### Feature extraction

Feature extraction is a crucial step in the protein identification process, which transforms the amino acid sequence of a protein into discrete data of a certain length and represents the protein sample with features composed of discrete data. It has been demonstrated that the physicochemical properties and evolutionary information of amino acids provide a more comprehensive picture of protein properties. Therefore, we extract AADP-PSSM features and the RPSSM features, which reflects the evolutionary information of proteins, for classification. For ease of representation, we firstly use the sigmoid function [17] to scale the PSSM elements to a range from 0 to 1. The AADP-PSSM and RPSSM are described in detail in the following two subsections.

#### Reduced position specificity score matrices (RPSSM)

The RPSSM [18] is obtained by merging PSSM based on amino acid similarity and then transforming the features with the autocovariance. This is calculated as follows.

Firstly, we simplify the original PSSM of LX20 to PSSM of LX10 based on amino acid similarity [19].

Secondly, the amino acid pseudo-composition is further obtained from the simplified PSSM, as shown in Eq. (1).

1 $$\begin{aligned} D_s= \frac{1}{L}\sum _{i=1}^L(p_{i,s}-\bar{p_s})^2\quad (\bar{p_s}=\sum _{i=1}^Lp_{i,s},s=1,2,\dots ,10;i=1,2,\dots ,L). \end{aligned}$$

where $p_{i,s}$ represents the pseudo-composition of amino acid $a_i$ when the amino acid $a_i$ is mutated to ’s’.

Subsequently, to partially reflect local sequence order effects, we extend the traditional dipeptide composition of protein sequences to RPSSM. All dipeptide pseudo-compositions in protein sequences are obtained, as defined in Eq. (2).

2 $$\begin{aligned} D{_s,_t}= \frac{1}{L-1}\sum _{i=1}^{L-1}x_{i,i+1}= \frac{1}{L-1}\sum _{i=1}^{L-1}\frac{(p{_i,_s}-{p_{i+1},_t})^2}{2} \quad (s,t=1,2,\dots ,10). \end{aligned}$$

where $x_{i,i+1}$ represents the pseudo-composition of the dipeptide $a_ia_{i+1}$ when amino acid $a_i$ is mutated to ’s’ and amino acid $a_{i+1}$ is mutated to ’t’.

Finally, 110-dimensional features are extracted from each query protein sequence.

#### AADP-position specificity score matrices (AADP-PSSM)

The feature is a combination of an amino acid combination and a dipeptide combination feature carrier, i.e. consisting of DPC-PSSM and ACC-PSSM.

The DPC-PSSM [20] is obtained by summing and averaging the product of the *i*th amino acid and the *j*th amino acid in two adjacent rows of PSSM, as shown in Eq. (3).

3 $$\begin{aligned}&y{_i,_j}= \frac{1}{L-1}\sum _{k=1}^{L-1}p{_k,_j}\times {p{_{k+1},_j}} \quad (1\le i,j\le 20). \end{aligned}$$

The ACC-PSSM [20] is obtained by averaging each column of the mapped PSSM, as shown in Eq. (4).

4 $$\begin{aligned} x_j= \frac{1}{L}\sum _{k=1}^{L-1}p{_i,_j} \quad (j=1,2,\dots ,20). \end{aligned}$$

where $x_j(1\le j\le 20)$ is the composition of amino acid *j*-types in PSSM and represents the average score of amino acid residues in protein S that have mutated to amino acid *j*-types over the course of evolution.

5 $$\begin{aligned} \mathbf {(x_1,\dots ,x_{20},y_{1,1},\dots ,y_{1,20},y_{2,1},\dots ,y_{2,20},\dots ,y_{20,20})}^\top . \end{aligned}$$

Finally, we obtain 420 dimensional features as shown in Eq. (5).

### Feature selection

In this study, we employ MRMD algorithm that proposed by Zou [21] for dimensionality reduction. The MRMD algorithm analyzes the contribution of each feature to the prediction process by focusing on two aspects: maximum correlation and maximum distance, i.e., maximizing the correlation between features and categorical variables, and minimizing the correlation between features and features. It takes into account not only the correlation between features and labels, but also the correlation between features and features. After dimensionality reduction by MRMD algorithm we get the sub-feature set with low redundancy and strong correlation with the target class.

### Ensemble model

**Fig. 2:**
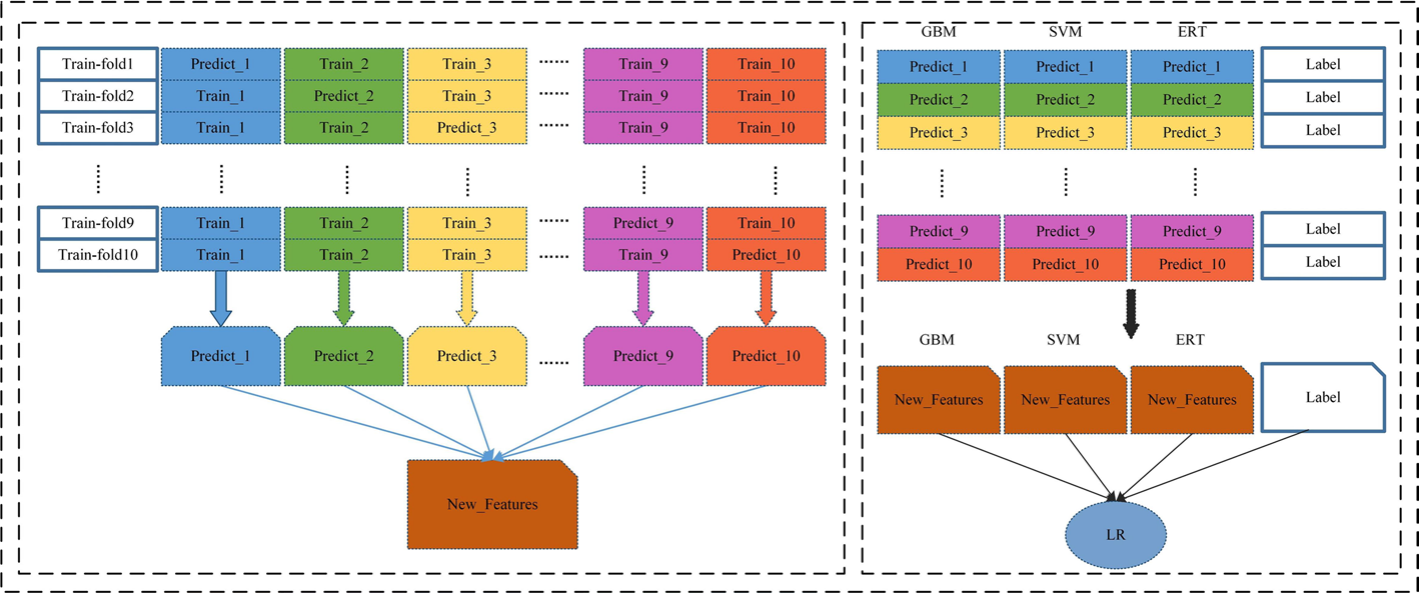
The Stacked ensemble classifier framework

Research shows that the application of embedded learning methods can improve the predictive performance of various bioinformatics applications [22, 23]. In this study, we construct a two-layer stacked ensemble classifier [24], and the framework of the stacked ensemble classifier is shown in Fig. 2. Before 10-fold cross-validation, we use the undersampling method to deal with the data imbalance problem of the original data set. Then, the undersampled training dataset is divided into 10 equal and non-repetitive parts, of which 1 part is used as the validation dataset and 9 parts as the training dataset, forming a combination of 10 sets of training and validation datasets. In the first layer, the 10 folds of data is fed into GBM, SVM and ERT to obtain the predicted values. In the second layer, the outputs from the three models are stitched together and fed into the logistic regression classifier to obtain the final prediction results.

### Classification algorithm

#### Extreme random trees

Extreme random trees (ERT) [25] is a machine learning algorithm that uses multiple trees to train and predict samples, proposed by Geurts P in 2006, which selects its cutpoints completely randomly for a given numerical attribute, i.e. independent of the target variable. At each tree node, a random selection of a certain number of attributes is combined and the best of these attributes is determined. In the extreme case, the method randomly selects individual attributes as cutpoints at each node, thus constructing a completely random tree whose structure is independent of the value of the target variable of the learning sample. By choosing the parameters appropriately, the strength of the randomization can be adjusted to the details of the problem. In this study, we set the number of trees in the ERT to 100, the minimum number of samples required to split internal nodes to 2, and the minimum number of samples required for leaf nodes to 1.

#### LightGBM

LightGBM (GBM) [26] is a lightweight (Light) gradient boosting machine (GBM), another evolutionary version of the GBDT model [27]. It employs two new techniques, Gradient-based One-Side Sampling (GOSS) and Exclusive Feature Bundling (EFB), to speed up the training process of traditional GBDT by more than 20 times without compromising accuracy. And it compensates for the limitations of the histogram-based algorithm. In order to make the model work positively, a grid search method is used to find the optimal parameters. We set the learning rate of GBM to 0.05, the number of base learners to 400, maximum depth of the tree to 7, and the number of subsamples to 0.8.

#### Support vector machine

Support vector machine (SVM) [28] is a supervised learning algorithm for classification with great robustness. SVM is widely used in classification, regression and other tasks [29, 30], as a generalized linear classifier that aims to find the maximum bounded hyperplane as the decision boundary to accomplish the classification task with great robustness. It achieves optimum performance mainly by adjusting two parameters, *C* and $\alpha$. *C* represents the penalty factor or tolerance, and the penalty accepted by the SVM in case of misclassification is positively correlated with *C*.$\alpha$ implicitly determines the distribution of the data once it is mapped to the new feature space; the larger the $\alpha$, the fewer the support vector. In order to find the best combination of parameters to make the model work positively, a grid search method is used to search for the optimal parameters. We set the kernel function of the SVM as a radial kernel function, the kernel function coefficient as 0.018, the penalty coefficient as 19.

#### Logistic regression

Logistic regression (LR) [31] is a generalized linear regression analysis model that is commonly used for binary classification. In binary classification, LR is linear regression with a sigmoid function (non-linear) mapping added to it to output discrete values. LR is sensitive around 0 and insensitive at locations far from 0. The model is more concerned with classification boundaries, which increases the robustness of the model. We choose lbfgs (Hessian matrices) to optimize the loss function optimization algorithm for LR, and the number of iterations of the optimization algorithm is set to 100.

### Evaluation metrics

A number of widely adopted evaluation metrics are used in this study, including accuracy (ACC), sensitivity (SN), specificity (SP), and mathews correlation coefficient (MCC). We also use the receiver operating characteristic (ROC) and the area under the curve (AUC) [32] to evaluate the performance of classifier. The evaluation metrics are expressed as follows.

6 $$\begin{aligned} SN= & {} \frac{TP}{TP+FN}. \end{aligned}$$

7 $$\begin{aligned} SP= & {} \frac{TN}{TN+FP}. \end{aligned}$$

8 $$\begin{aligned} ACC= & {} \frac{TP+TN}{TP+TN+FP+FN}. \end{aligned}$$

9 $$\begin{aligned} MCC= & {} \frac{TP\times TN-FP\times FN}{\sqrt{(TP+FN)(TP+FP)(TN+FP)(TN+FN)}}. \end{aligned}$$

10 $$\begin{aligned} AUC= & {} \frac{1}{2}(\frac{TP}{TP+FN}+ \frac{TN}{TN+FP}). \end{aligned}$$

where *TP*, *TN*, *FP* and *FN* indicate the rates of true positive, true negative, false positive and false negative, respectively.

## Results and discussion

### Comparison of different feature extraction methods

**Fig. 3:**
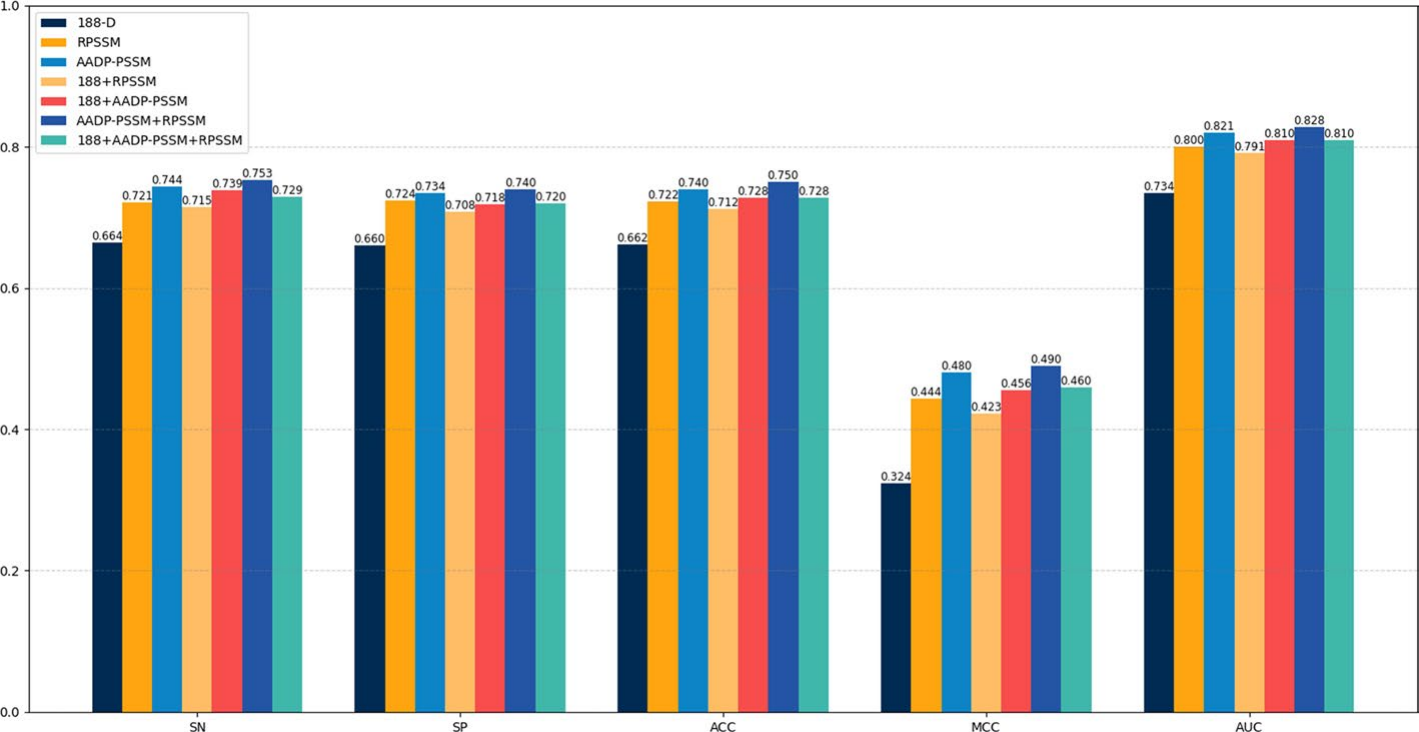
Comparison of different feature extraction methods

Since both the physicochemical properties and evolutionary information are important for protein prediction [33], we choose to compare the 188 features, which represent physicochemical properties, with the RPSSM and AADP-PSSM features, which represent evolutionary information. In this experiment, the univariate principle is used and only the method of feature extraction is changed to observe its effect on the experimental results. 10-fold cross-validation is used to evaluate our model and the results are shown in Fig. 3.

As shown in Fig. 3, when single features are compared, the classification accuracy of the RPSSM and AADP-PSSM features are 6.4% and 7.8% higher than the 188 features, respectively. Furthermore, all other evaluation metrics are also higher than that of the 188 features. When the two features are fused together for comparison, The combination of RPSSM and AADP-PSSM yields the best performance with an accuracy of 74.48%. When all three features are fused together for comparison, the results show that there is no significant improvement in the evaluation metrics. Finally, we choose RPSSM and AADP-PSSM as features to identify vesicle transport proteins.

### Base classifier selection

**Fig. 4:**
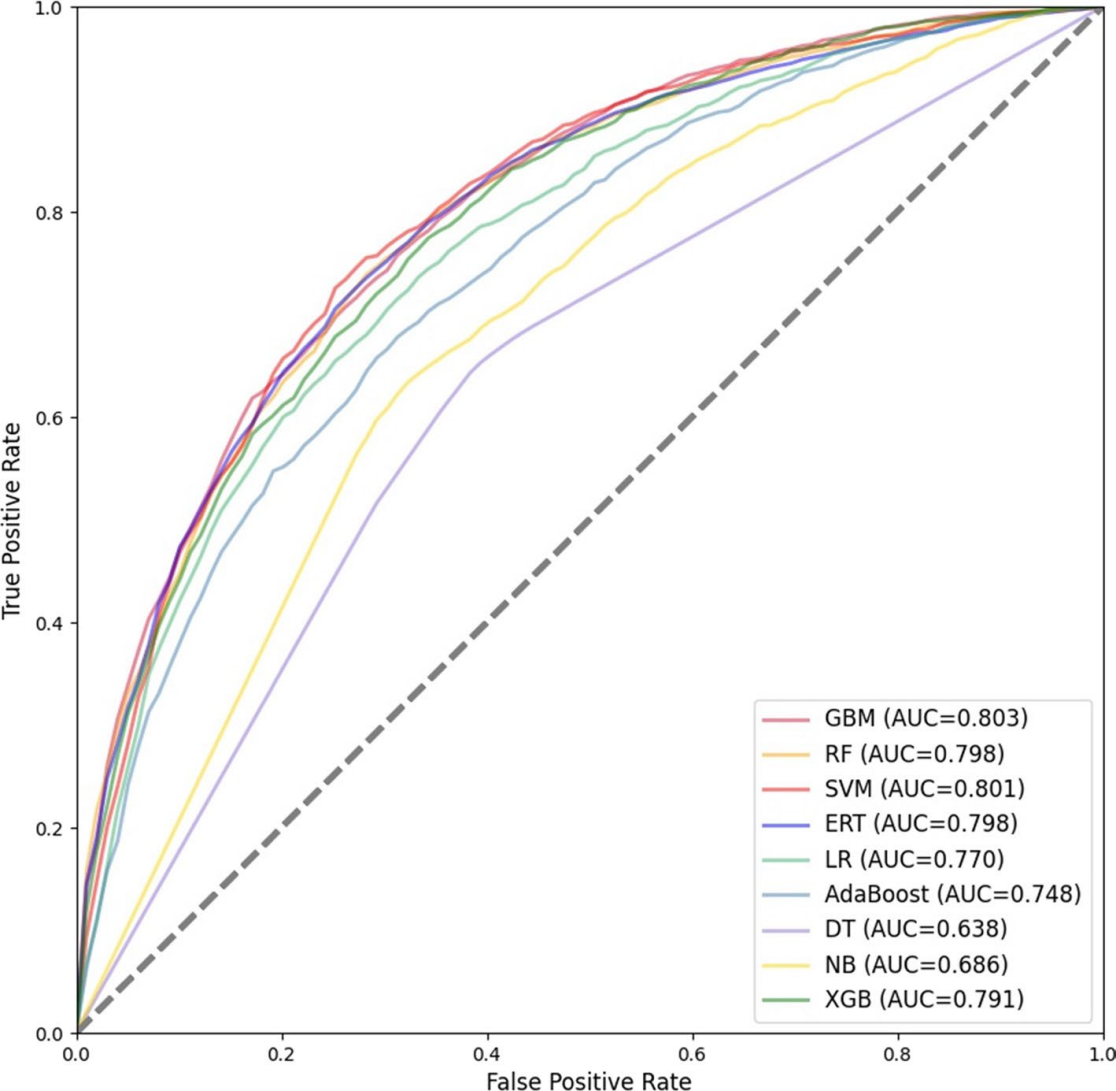
ROC curves for 10-fold cross-validation of independent classifiers

**Fig. 5:**
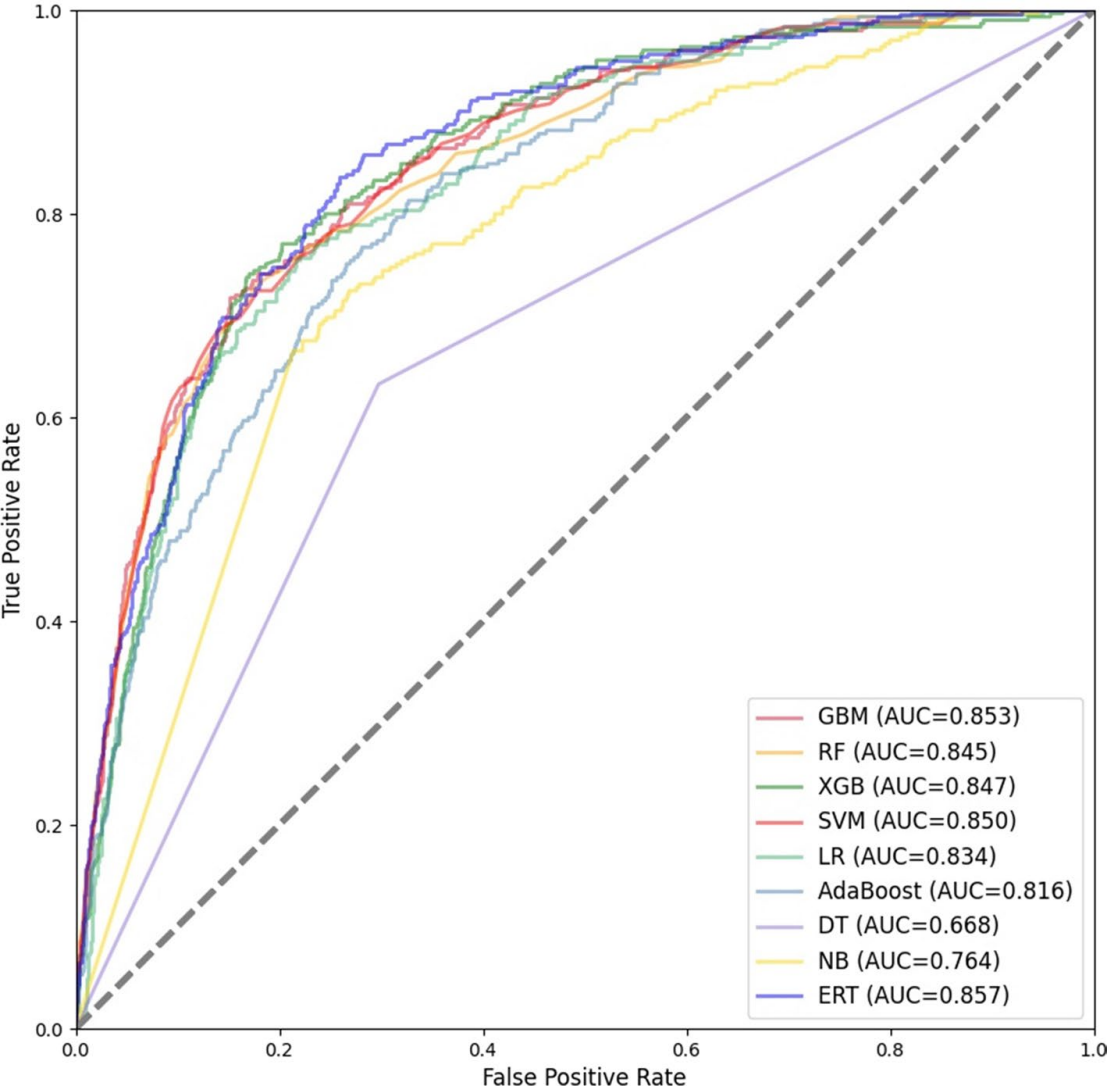
ROC curves of independent classifiers on testing set

Traditional machine learning classifiers have excellent stability and validity. Therefore, to determine the most suitable combination of base classifiers, we test nine traditional machine learning classifiers. The performance of the nine traditional machine learning classifiers on the training set with 10-fold cross-validation is shown in Table 2 and Fig. 4, and on the testing set is shown in Table 3 and Fig. 5.

**Table 2:** Performance of traditional classifiers on the training set with 10-fold cross-validation

	ACC (%)	SN	SP	MCC	Time	ACC_Std	SN_std	SP_Std	MCC_Std	AUC_Std
GBM	72.13	0.731	0.712	0.442	35.879	0.020	0.030	0.015	0.042	0.018
RF	72.66	0.719	0.736	0.454	44.149	0.021	0.038	0.013	0.041	0.019
SVM	73.06	0.752	0.711	0.463	181.59	0.014	0.029	0.023	0.031	0.014
ERT	72.47	0.731	0.720	0.450	13.433	0.023	0.033	0.018	0.045	0.020
LR	69.71	0.719	0.677	0.396	0.406	0.029	0.039	0.032	0.061	0.029
AdaBoost	67.82	0.689	0.668	0.357	76.94	0.024	0.034	0.025	0.048	0.017
DT	63.87	0.651	0.625	0.277	16.209	0.026	0.026	0.048	0.051	0.025
NB	63.98	0.721	0.559	0.282	2.220	0.020	0.044	0.013	0.042	0.022
XGB	71.43	0.721	0.707	0.428	57.816	0.012	0.015	0.019	0.022	0.018

${}^{\textrm{ACC}\_\textrm{Std,SN}\_\textrm{std,SP}\_\textrm{Std,MCC}\_\textrm{Std,AUC}\_\textrm{Std}}$ these are the standard deviations of ACC, SN, SP, MCC and AUC when each classifier performs 10-fold cross validation on the training set

As shown in Table 2, the ACC, SN and MCC of SVM are the highest on the training set over 10-fold cross-validation, which are 73.06%, 0.752 and 0.463, respectively. The ACC of RF and ERT are 0.4% and 0.59% lower than that of SVM, respectively. GBM and ERT achieve the same SN, and their SN are 0.731, which is the second highest among all classifiers. When performing 10-fold cross-validation, the classifier with the highest SP is RF, ERT achieves the second highest SP among all classifiers. We also evaluate the stability of classifiers by the standard deviation of each evaluation metric when performing 10-fold cross-validation. XGB has the lowest standard deviation in ACC, SN and MCC. SVM has the lowest standard deviation in AUC, second only to XGB in ACC, SN, SP. Considering the standard deviation of each evaluation metric, SVM and XGB have excellent stability.

**Table 3:** Performance of traditional classifiers on the testing set

	ACC (%)	SN	SP	MCC	Time
GBM	82.21	0.741	0.815	0.465	4.044
RF	80.32	0.740	0.815	0.465	5.097
SVM	80.71	0.757	0.817	0.480	23.873
ERT	81.06	0.762	0.821	0.491	1.528
NB	70.34	0.744	0.695	0.460	0.049
AdaBoost	75.36	0.708	0.762	0.380	9.083
DT	68.89	0.675	0.692	0.285	2.062
LR	77.87	0.741	0.786	0.431	0.145
XGB	79.99	0.756	0.808	0.467	6.740

As shown in Table 3, ERT performs the best on the testing set. The ACC, SN, SP and MCC of ERT are 81.06%, 0.762, 0.821 and 0.491, respectively. The SN, SP and MCC of ERT are the highest among all classifiers, and the ACC of ERT is second only to GBM. The SN, SP and MCC of SVM are 0.757, 0.817 and 0.480, respectively, which is the second highest among all classifiers, lower than that of ERT. DT performs the worst on the testing set. In terms of time, it takes a long time for SVM and XGB to perform 10-fold cross-validation on the training set and test on the testing set.

As shown in Fig. 4, the average AUC for 10-fold cross-validation of GBM, RF, SVM, RT, LR, AdaBoost, DT, NB and XGB on the training set are 0.803, 0.798, 0.801, 0.770, 0.748, 0.638, 0.686 and 0.791, respectively. The top four classifiers with the highest average AUC are GBM, SVM, RF and ERT. As shown in 5, the AUC of GBM, RF, XGB, SVM, LR, AdaBoost, DT, NB and ERT on the testing set are 0.853, 0.845, 0.847, 0.850, 0.834, 0.816, 0.668, 0.764 and 0.857, respectively. The top three classifiers with the highest AUC are ERT, GBM and SVM.

Combining all base classifiers in a single meta-classifier does not lead to optimal prediction performance, so searching for the optimal combination of base classifiers is required. Taking classifier performance into account, GBM uses paralleleaf splitting to generate more complex trees than horizontal splitting, which can achieve higher accuracy. SVM can achieve great performance with a solid theoretical foundation and great robustness. The best bifurcation property of ERT is randomly selected, with excellent performance in vesicle transport proteins recognition. Finally, we choose a combination of SVM, GBM and ERT to construct the base classifier and perform experiments, finding that the combination of these three classifiers performs the best.

### Meta-classifier selection

**Fig. 6:**
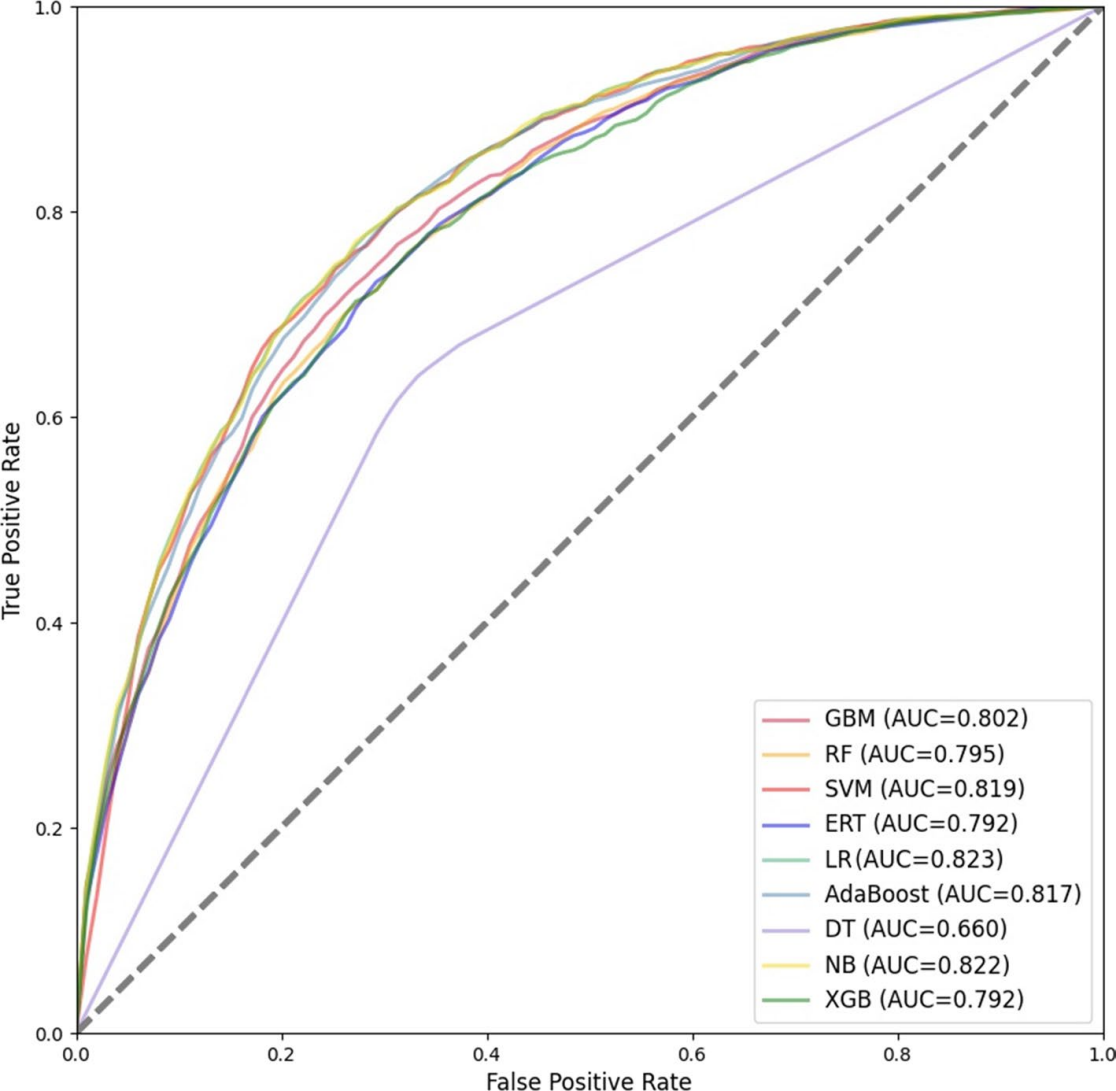
ROC curves for 10-fold cross-validation of different meta-classifiers on the training set

**Fig. 7:**
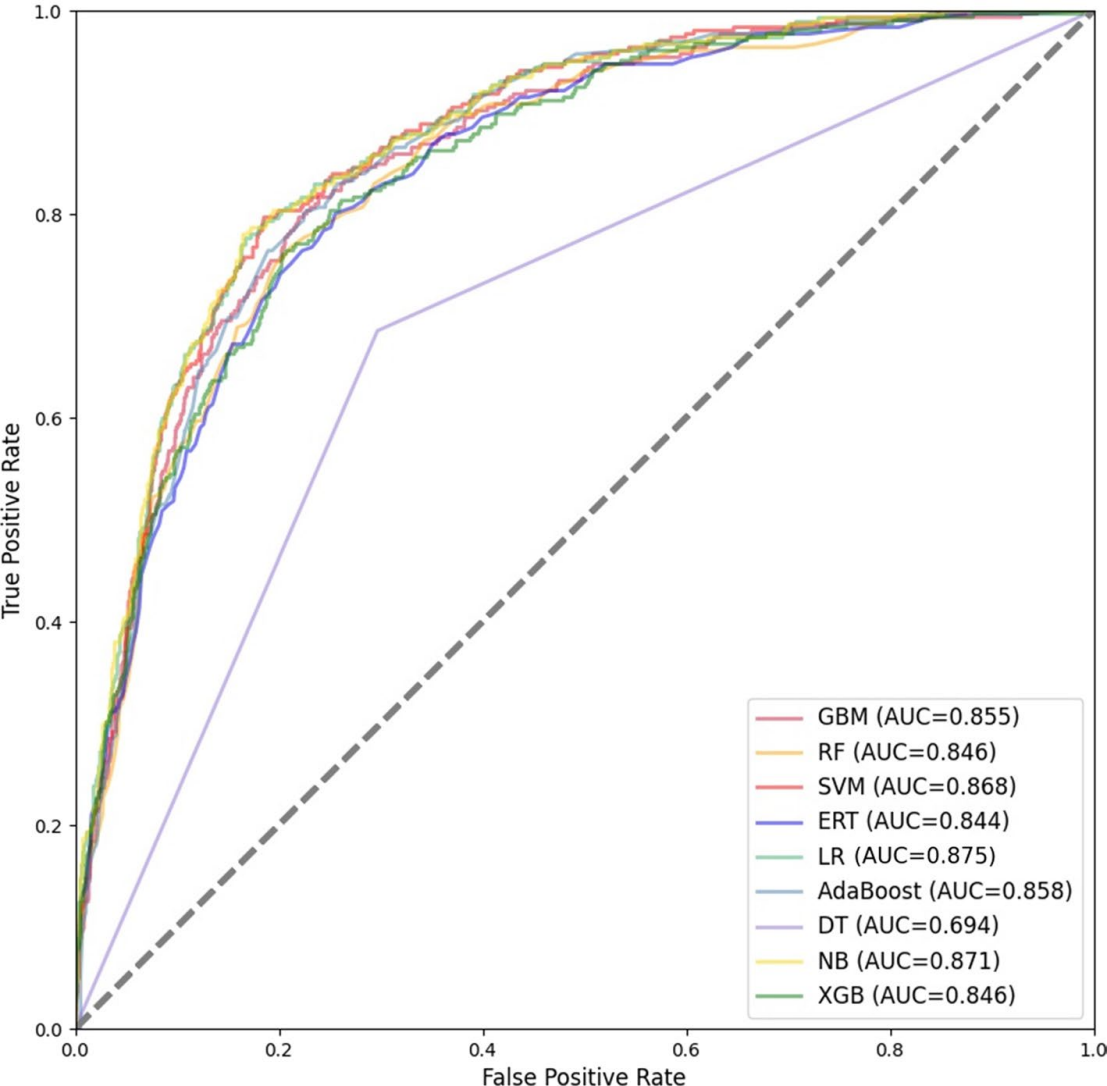
ROC curves of different meta-classifiers on testing set

After the first stage of training, we determine the optimal base classifier combination is SVM, GBM, ERT. In the second stage, we feed the output of each base classifier as new features of the protein sequence into the meta-classifier for training to obtain the final result. Therefore, the choice of meta-classifier also plays an important role in the building of the stacked model. In this study, in order to determine the meta classifiers, we combine 9 traditional machine learning classifiers as meta-classifiers with the above selected classifier combination, 9 stacked ensemble classifiers are constructed. The results of 10-fold cross-validation on the training set are shown in Table 4 and Fig. 6, and the performance on the testing set is shown in Table 5 and Fig. 7.

**Table 4:** Performance of different meta-classifiers on the training set with 10-fold cross-validation

	ACC (%)	SN	SP	MCC	Time	ACC_Std	SN_std	SP_Std	MCC_Std	AUC_Std
GBM	72.92	0.734	0.726	0.459	382.590	0.014	0.026	0.023	0.027	0.018
RF	71.82	0.716	0.722	0.437	277.694	0.016	0.025	0.026	0.032	0.018
SVM	73.66	0.796	0.679	0.478	287.667	0.015	0.041	0.036	0.033	0.019
ERT	72.10	0.715	0.729	0.443	365.482	0.015	0.026	0.017	0.030	0.017
NB	74.50	0.754	0.737	0.490	365.941	0.016	0.020	0.024	0.033	0.015
AdaBoost	74.27	0.754	0.732	0.485	366.721	0.017	0.027	0.018	0.035	0.014
DT	64.75	0.651	0.645	0.295	364.414	0.020	0.031	0.030	0.039	0.020
LR	74.90	0.760	0.738	0.498	351.539	0.018	0.023	0.020	0.035	0.015
XGB	72.01	0.726	0.716	0.441	367.720	0.017	0.019	0.029	0.035	0.018

${}^{\textrm{ACC}\_\textrm{Std,SN}\_\textrm{std,SP}\_\textrm{Std,MCC}\_\textrm{Std,AUC}\_\textrm{Std}}$ these are the standard deviations of ACC, SN, SP, MCC and AUC when each classifier performs ten-fold cross validation on the training set

**Table 5:** Performance of different meta-classifiers on the testing set

	ACC (%)	SN	SP	MCC	Time
GBM	80.16	0.744	0.813	0.450	244.699
RF	78.04	0.741	0.788	0.433	250.098
SVM	79.88	0.803	0.798	0.491	253.355
ERT	78.99	0.727	0.803	0.440	252.610
NB	79.97	0.774	0.805	0.481	253.355
AdaBoost	78.21	0.798	0.779	0.464	252.061
DT	70.23	0.702	0.702	0.315	252.058
LR	81.33	0.768	0.823	0.499	258.759
XGB	78.32	0.731	0.794	0.432	249.503

As shown in Table 4, the ensemble model performs best on the training set when LR is used as a meta-classifier, and it achieves the highest ACC, SP, and MCC of 74.90%, 0.738 and 0.498, respectively. The ensemble model performs second only to LR when NB is used as a meta-classifier. As shown in Table 5, ranked in descending order of accuracy, the results of each classifier as a meta-classifier on the testing set are LR, GBM, NB, SVM, ERT, XGB, AdaBoost, RF, DT. When SVM, NB and LR are used as meta-classifiers respectively, the ensemble model has a higher SN on the testing set, and the SN are 0.803,0.774 and 0.768 respectively. The SP and MCC of LR as a meta-classifier on the testing set are higher than other classifiers.

We further use the ROC curve to evaluate the performance of the ensemble model for different meta-classifiers. When LR is used as the meta-classifier, the ROC curve covers the largest area for 10-fold cross validation and testing on the independent testing set. The AUC of LR as a meta-classifier is 0.823 when performing 10-fold cross-validation, and the AUC is 0.875 when testing on the testing set. The AUC of NB as a meta-classifier is close to that of LR, with a gap of 0.001 in 10-fold cross-validation and 0.004 on the testing set. Considering all the evaluation metrics, the ensemble model has the best results with the LR selected as the meta classifier. Therefore, we finally construct a two-layer stacked model with LR as the meta-classifier and SVM, ERT, GBM as the base classifier to identify vesicle transport proteins.

### Comparison of different dimensionality reduction methods

**Fig. 8:**
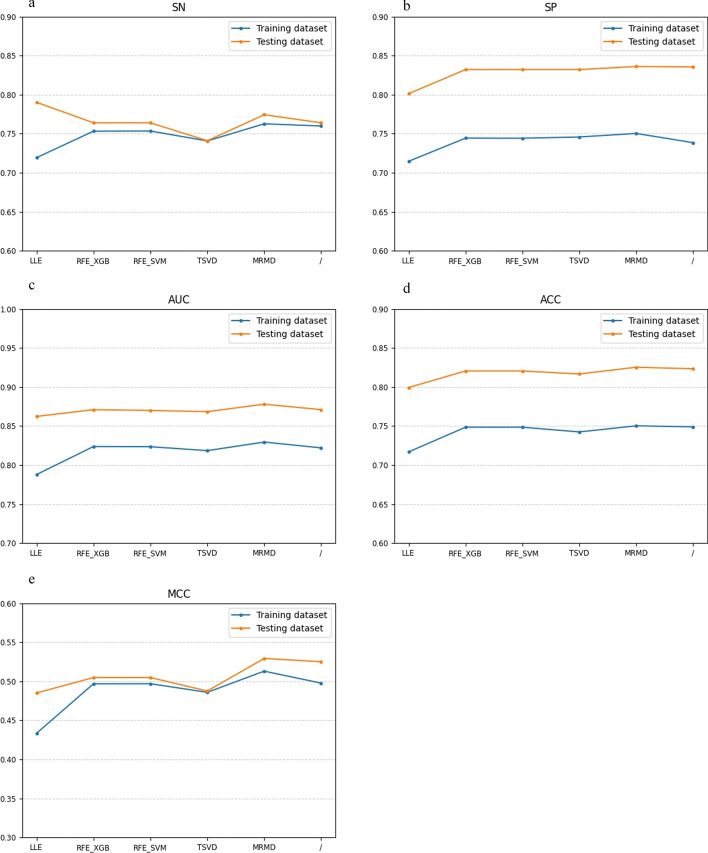
Performance of different dimensionality reduction methods on the training and testing set

In the machine learning process, a high dimensionality of the input feature vector will make the model excessively complex and reduce the generalization ability of the model, so we need to reduce the dimensionality of the features to enhance the performance of the model. We test five different feature selection methods, SVM-RFE [34], TSVD [35], local linear embedding (LLE) [36], MRMD and XGB-RFE [37], to reduce the dimension of our features, of which dimension is 530. A stacked ensemble classifier is used to classify the optimal subset of features obtained by different dimensionality reduction methods. The result is shown in Fig. 8.

After dimension reduction by SVM-RFE, TSVD, LLE, MRMD and XGB-RFE, the size of sample space is similar. As shown in Fig. 8 (a-e), among the five dimensionality reduction methods, MRMD achieve better results than the other methods in each evaluation metrics of SN, SP, ACC, MCC and AUC.

### Comparison with other methods

Through the above processing, we obtain a good performance model. In order to evaluate the predictive ability of our model more fairly and objectively. In this study, considering the influence of data on the experimental results, we use the same data preprocessing method to process the data. we evaluate the performance of our model by comparing it with the typical vesicle transport proteins identification methods on the testing set, and with the ensemble model proposed by Chen. The methods mentioned above are shown in Table 6. In order to further verify that the stacking method is an ensemble strategy suitable for accurately identifying vesicle transport proteins, we also test two types of majority voting methods based on GBM, SVM and ERT on testing set. The results are presented in Table 7.

**Table 6:** Details of the previous method

Researcher	Feature	Feature select method	Model
Nguyen Quoc Khanh Le	PSSM	\	CNN+GRU
Tao	CDCT	MRMD	Libsvm
Gong	CSP-SegPseP-SegACP	MRMD	XGBoost
Chen	SVMProt-188D	LDA	Bagging-SVM

“\” indicating that the study did not use feature selection method

**Table 7:** Performance comparison with other methods on independent testing set

	SN	SP	ACC (%)	MCC	AUC
Libsvm-MRMD [9]	0.722	0.725	72.16	0.342	0.796
VTP-Identifier [10]	0.761	0.829	81.77	0.499	0.876
GRU [8]	0.708	0.829	80.91	0.459	0.850
bagging-SVM [15]	0.734	0.817	80.32	0.463	0.854
Hard voting	0.769	0.815	80.43	0.467	0.864
Soft voting	0.774	0.817	80.94	0.492	0.969
Stack-VTP	0.774	0.836	82.53	0.521	0.889

As shown in Table 7. The ACC, MCC, SP and SN of the our proposed stacked classifier are the best, which are 82.53%, 0.521, 0.836 and 0.774, respectively. Compared with hard voting and soft voting ensemble strategy, the ACC, SP and MCC are higher than that of hard voting and soft voting 1.54%, 1.59% and 0.021, 0.019 and 0.054, 0.029, respectively. Compared with the recently proposed bagging-SVM ensemble method, the SN, SP, MCC, ACC and AUC of our method are 0.04, 0.019, 2.21%, 0.058 and 0.035 higher, respectively. Compared with the typical methods for predicting vesicle transport proteins, the performance of our model is better than the existing methods, and the ACC is 10.37%, 1.62% and 0.76% higher than that of Libsvm-MRMD, GRU and VTP-Identifier, respectively.

In order to further evaluate our proposed method. McNemar’s test [38] is used to test whether any difference in performance between two classification methods that test on the same dataset is statistically significant. This study compares our proposed method with each other, and the joint performance of the two methods can be summarized as a 2 $\times$ 2 contingency table. The contingency table contains the number of samples correctly classified by the two methods, the number of samples not correctly classified by the two methods, only the number of samples correctly classified by the first method, and the number of samples correctly classified by the second method. It is assumed that the two methods have the same error rate. Finally, the hypothesis is verified by *p*-value, and we get $p<0.05$. The obtained results show that the proposed approach outperforms all other commonly used methods.

## Conclusion

This study proposes a stacked ensemble model to identify vesicle transport proteins. Firstly, we choose a combination of RPSSM and AADP-PSSM features. Secondly, the imbalanced data are preprocessed by undersampling and MRMD is applied to select the optimal subset of features. Finally, a two-layer stacked model with GBM, SVM and ERT as base classifiers and LR as a meta-classifier is constructed. On the independent testing set, The SN, SP, ACC and MCC are 0.774, 0.836, 82.53%, 0.521, respectively. Comparing the model proposed in this study with existing machine learning based models, the experimental results show that the accuracy (ACC), SN, SP and MCC of our proposed model are 0.76%, 0.013, 0.007 and 0.013 higher than the current state-of-the-art models, respectively. In summary, the proposed model perform better in the field of Vesicle transport proteins identification than other state-of-the-art models, proving the effictiveness of our model. The method is expected to be an effective bioinformatics tool for the identification of vesicle transport proteins.

Although our method has achieved certain success, it still has limitations. The limitation of not having a large and single sample size of data prevents us from deeply exploring the relationship between vesicle transport proteins and other transport proteins. And we lack a user-friendly and publicly available web server to facilitate the use of researchers. We hope we will be able to build a more effective dataset containing multiple transport proteins to facilitate in-depth exploration of the connections between transporter proteins in future studies. And we hope to provide a web server for the proposed method in this paper. If our future research achieve success, it will lead to great progress in the field of transport protein research.

## Data Availability

The data that support the findings of this study are available from Le et al. [8] but restrictions apply to the availability of these data, which were used under license for the current study, and so are not publicly available. Data are however available from the authors upon reasonable request and with permission of Le et al.
